# Supplementary figures and images for: Modeling vaccination strategies in an Excel spreadsheet: Increasing the rate of vaccination is more effective than increasing the vaccination coverage for containing COVID-19
Source: PLoS One. 2021 Jul 19;16(7):e0254430. doi: 10.1371/journal.pone.0254430 (PMC8289062; doi:10.1371/journal.pone.0254430)

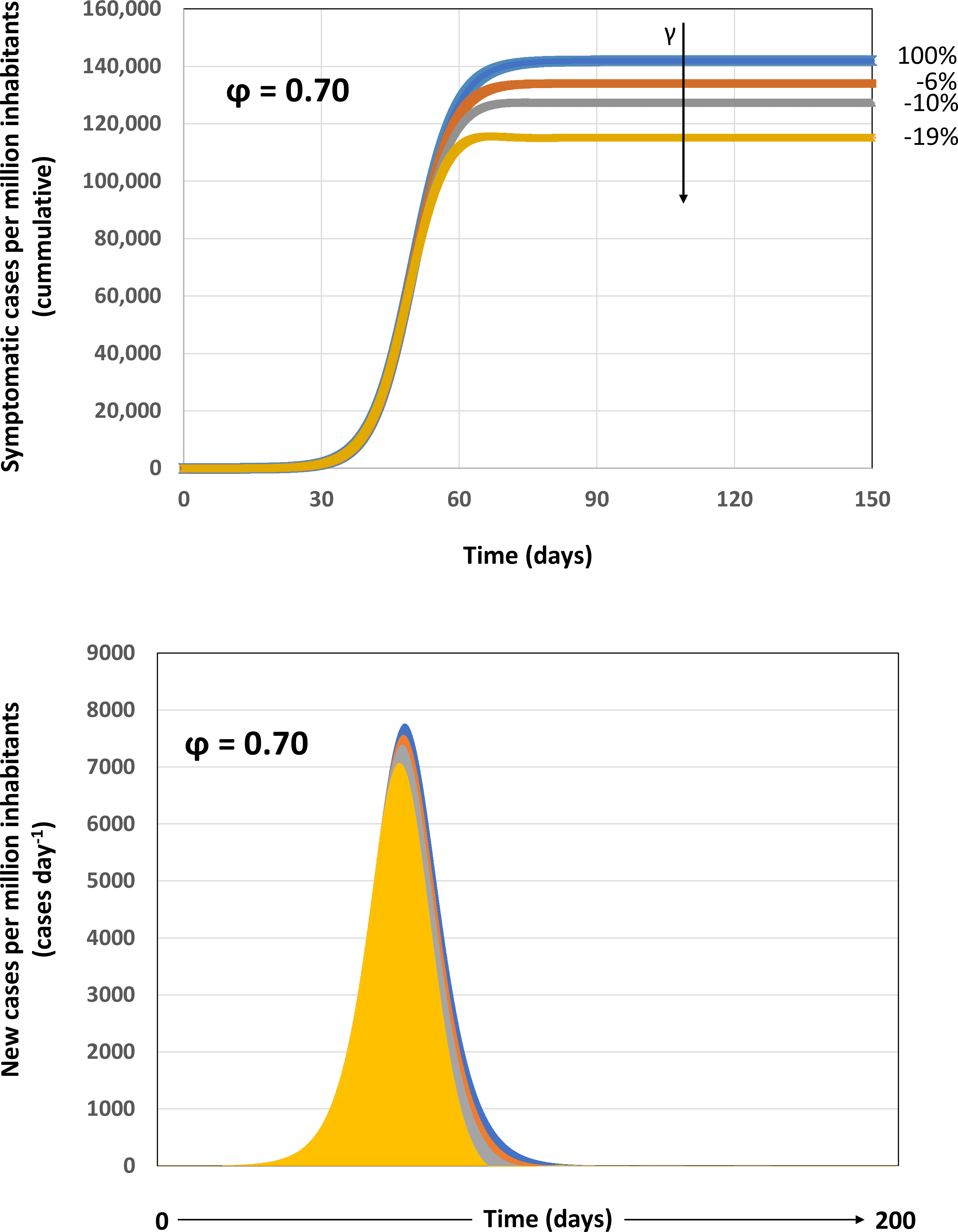

Supplement: S1 Fig — (A) The cumulative number of infections, (B) the number of new infections per day and the maximum bed occupancy (inset) are presented for vaccination scenarios in which the vaccine coverage is kept constant at 70% Po (φ = 0.70), the effectiveness of the social distancing measures is 20% (σ = 0.20), a basal level of testing is established (α = 0.15), and different vaccination rates are imposed, such that: the entire population could be vaccinated within a year (Po year-1; orange curve), within six months (2 Po year-1; grey curve), or within three months (vaccination at 4 Po year-1; yellow curve). A reference scenario without vaccination is included (blue line). Numbers indicate the percentage of reduction of symptomatic cases that results from the application of each vaccination rate with respect to the reference case. (TIFF) [file pone.0254430.s002.tiff]
