# Supplementary material for: Modeling vaccination strategies in an Excel spreadsheet: Increasing the rate of vaccination is more effective than increasing the vaccination coverage for containing COVID-19
Source: PLoS One. 2021 Jul 19;16(7):e0254430. doi: 10.1371/journal.pone.0254430 (PMC8289062; doi:10.1371/journal.pone.0254430)
Supplement: S1 Table — The intrinsic rate of infection ranges between 0.30 and 0.37 for territories with population densities between 4,000 and 7,000 hab km2. (DOCX) [file pone.0254430.s003.docx]

**Modeling vaccination strategies in an Excel spreadsheet:** **Increasing the rate of vaccination is more effective than increasing the vaccination coverage for containing COVID-19**

**S1 Table**

Mario Moisés Alvarez^1,2*^, Sergio Bravo-González^1,2^, and Grissel Trujillo-de Santiago^1,3^

^1^ Centro de Biotecnología-FEMSA, Tecnologico de Monterrey, Monterrey 64849, NL, México

^2^ Departamento de Bioingeniería, Escuela de Ingeniería y Ciencias, Tecnologico de Monterrey, Monterrey 64849, NL, México

^3^ Departamento de Ingeniería Mecatrónica y Eléctrica, Escuela de Ingeniería y Ciencias, Tecnologico de Monterrey, Monterrey 64849, NL, México

(*) corresponding author: [*mario.alvarez@tec.mx*](mailto:mario.alvarez@tec.mx)

**S1 Table.** Specific infection rates (µ_o_) and the associated doubling times (t_d_) for COVID-19 infection in different geographic regions. Note that the intrinsic rate of infection ranges between 0.30 and 0.37 for territories with population densities between 4,000 and 7,000 hab km^2^.

| **Territory** | **Population density** | **µ** | **t_d_** |
| --- | --- | --- | --- |
|  | (hab km^2^) | (day^-1^) | (day) |
| Sao Paulo; Brazil | ~7,000 | 0.308 | 2.250 |
| Mexico City; México | 6,000 | 0.329 | 2.107 |
| Toronto; Canada | 4,149 | 0.330 | 2.100 |
| Lisbon; Portugal | 5,476 | 0.341 | 2.032 |
| Madrid; Spain | 5,418 | 0.358 | 1.937 |
| London; England | 5,701 | 0.362 | 1.915 |
| Amsterdam; Netherlands | 4,439 | 0.424 | 1.635 |
| NYC; USA | 10,194 | 0.666 | 1.040 |
