# Supplementary material for: Modeling vaccination strategies in an Excel spreadsheet: Increasing the rate of vaccination is more effective than increasing the vaccination coverage for containing COVID-19
Source: PLoS One. 2021 Jul 19;16(7):e0254430. doi: 10.1371/journal.pone.0254430 (PMC8289062; doi:10.1371/journal.pone.0254430)
Supplement: S2 Table — Indicators are calculated for different vaccination scenarios, including the day of the epidemic peak, the number of new infection cases at the epidemic peak, the cumulative number of symptomatic infections after 150 days of the local pandemic onset, the maximum bed occupancy, and the number of fatalities at different case fatality rates. (DOCX) [file pone.0254430.s004.docx]

**Modeling vaccination strategies in an Excel spreadsheet:** **Increasing the rate of vaccination is more effective than increasing the vaccination coverage for containing COVID-19**

**S2 Table**

Mario Moisés Alvarez^1,2*^, Sergio Bravo-González^1,2^, and Grissel Trujillo-de Santiago^1,3^

^1^ Centro de Biotecnología-FEMSA, Tecnologico de Monterrey, Monterrey 64849, NL, México

^2^ Departamento de Bioingeniería, Escuela de Ingeniería y Ciencias, Tecnologico de Monterrey, Monterrey 64849, NL, México

^3^ Departamento de Ingeniería Mecatrónica y Eléctrica, Escuela de Ingeniería y Ciencias, Tecnologico de Monterrey, Monterrey 64849, NL, México

(*) corresponding author: [*mario.alvarez@tec.mx*](mailto:mario.alvarez@tec.mx)

**S2 Table.** Effect of different vaccination scenarios in relevant indicators of the local evolution of pandemic COVID-19. Indicators are calculated for different vaccination scenarios, including the day of the epidemic peak, the number of new infection cases at the epidemic peak, the cumulative number of symptomatic infections after 150 days of the local pandemic onset, the maximum bed occupancy, and the number of fatalities at different case fatality rates.

**Table S2.** (continuation) Effect of different vaccination scenarios in relevant indicators of the local evolution of pandemic COVID-19.

**Table S2.** (continuation) Effect of different vaccination scenarios in relevant indicators of the local evolution of pandemic COVID-19.
